# Alterations in the human gut microbiome associated with *Helicobacter pylori* infection

**DOI:** 10.1002/2211-5463.12694

**Published:** 2019-08-10

**Authors:** Daoming Wang, Yan Li, Huanzi Zhong, Qiuxia Ding, Yuxiang Lin, Shanmei Tang, Yang Zong, Qi Wang, Xiuqing Zhang, Huanming Yang, Rong Wang, Xiao Liu

**Affiliations:** ^1^ School of Future Technology University of Chinese Academy of Sciences Beijing China; ^2^ BGI‐Shenzhen China; ^3^ China National GeneBank BGI‐Shenzhen China; ^4^ Laboratory of Genomics and Molecular Biomedicine Department of Biology Copenhagen Biocenter University of Copenhagen Denmark; ^5^ iCarbonX Shenzhen China; ^6^ James D. Watson Institute of Genome Sciences Hangzhou China; ^7^ Icahn Institute and Department of Genetics and Genomic Sciences Icahn School of Medicine at Mount Sinai New York NY USA

**Keywords:** gut microbiome, *Helicobacter pylori*, infection, metagenome, vitamin B12 deficiency

## Abstract

*Helicobacter pylori* infection (HPI) is a prevalent infectious disease associated with gastric ulcer, gastric cancer, and many nongastrointestinal disorders. To identify genes that may serve as microbial markers for HPI, we performed shotgun metagenomic sequencing of fecal samples from 313 Chinese volunteers who had undergone a C14 breath test. Through comparing differences in intestinal microbial community structure between *H. pylori*‐positive and *H. pylori*‐negative individuals, we identified 58 HPI‐associated microbial species (*P* < 0.05, Wilcoxon test). A classifier based on microbial species markers showed high diagnostic ability for HPI (AUC = 0.84). Furthermore, levels of gut microbial vitamin B12 (VB12) biosynthesis and plasma VB12 were significantly lower in *H. pylori*‐positive individuals compared with *H. pylori*‐negative individuals (*P* < 0.05, Wilcoxon test). This study reveals that certain alterations in gut microbial species and functions are associated with HPI and shows that gut microbial shift in HPI patients may indirectly elevate the risk of VB12 deficiency.

**Abbreviations**
AUC: area under the receiver operating curve
BMI: body mass index
HPI: *Helicobacter pylori* infection
IGC: integrated gene catalog
IMG: integrated microbial genomes and microbiomes database
KEGG: Kyoto encyclopedia of genes and genomes
LC‐MS/MS: liquid chromatography–tandem mass spectrometry
MLG: metagenomics linkage group
PCoA: principal coordinate analysis
RA: rheumatoid arthritis
ROC: receiver operating curve
VB12: vitamin B12

*Helicobacter pylori*, a microaerophilic Gram‐negative bacterium, was first isolated in 1984 from patients with chronic active gastritis 1. *H. pylori* infection (HPI) is a prevalent infectious disease with a global prevalence of 44.3% 2 and can lead to gastrointestinal diseases such as peptic ulcer, atrophic gastritis, and gastric carcinoma 3. Although *H. pylori* predominantly colonizes the stomach, previous epidemiological studies have reported that HPI correlates with certain extragastric diseases 4, including vitamin B12 (VB12) deficiency 5, 6, 7, iron deficiency anemia 8, inflammatory bowel disease 9, and even neurodegenerative diseases 4.

Several previous studies have revealed that HPI correlates with a shift in gastrointestinal microbiota 10, 11, 12, 13, immune responses 14, and metabolic imbalances of host 6, 7. The microbiota of Mongolian gerbil gastrointestinal tracts were distinctly altered after long‐term infection with the *H. pylori* B8 wild‐type strain, with the luminal loads of *Escherichia coli* and enterococci in the cecum and *Bacteroides*/*Prevotella* spp. in the colon strikingly elevated 10. In mice, continuous HPI alters the gastric and intestinal microbiota community structure. Additionally, the infection causes increased expression of immune response‐related genes in gastric and pulmonary tissues 13. In humans, the relative abundances of dominant phyla in the gut of HPI‐positive individuals, including *Bacteroidetes*,* Firmicutes*, and *Proteobacteria,* are significantly different from HPI‐negative individuals and may correlate with gastric lesions 12. Usually, alterations in the microbiome are accompanied by differences in microbial functions; the relative abundance of 19 predicted gut microbial pathways is significantly different between *H. pylori*‐positive and *H. pylori*‐negative individuals 11. Besides the effect on host gastrointestinal microbes, persistent infection with *H. pylori* can cause detrimental inflammatory processes 14, and epidemiological studies have revealed that HPI correlates with lower VB12 levels in the blood 6, 7.

Previous HPI‐related intestinal microbiome studies used 16S rRNA amplicon sequencing, which may be limiting because of the inadequate resolution for microbial taxonomy and function 15. In addition, primer selection and PCR amplification can introduce bias when quantifying taxa abundance 16. Therefore, it remains unclear how many bacterial genes and species could serve as microbial markers for HPI and how a shift in gut microbiome alters the host's metabolism and immune responses.

In this study, we performed a shotgun metagenomic sequencing of fecal samples from 313 Chinese volunteers who had undergone a C14 breath test. We compared the differences in intestinal microbial community structure between *H. pylori*‐positive and *H. pylori*‐negative individuals and identified the gut microbial species and functions associated with HPI. In addition, the blood metabolite data of the 313 volunteers were collected. To further investigate how HPI influences host health, we analyzed the alterations of blood metabolite levels to explore the potential physiological effects of HPI mediated by gut microbes.

## Materials and methods

### Study samples

Fresh stool samples for the metagenomic sequencing were obtained from volunteers recruited in the Yantian District, Shenzhen, China. The C14 breath test to determine HPI status and other blood biochemistry level assessments were performed in a local hospital, and plasma VB12 levels were quantified using LC‐MS/MS (Table S1). None of the volunteers had taken any antibiotics within the previous 3 months. In addition, volunteers with serious illnesses, metabolic diseases, and pregnancies were excluded from the study. The samples were divided into *H. pylori*‐positive and *H. pylori*‐negative groups according to the C14 test results. The summary statistics of the study samples are provided in Table 1.

**Table 1:** Basic information of study volunteers. F, females; M, males

	All	Positive	Negative
Number	313 (148 F, 165 M)	128 (59 F, 69 M)	185 (89 F, 96 M)
Age	20–66 (28.14)	20–44 (27.77)	21–66 (28.39)
BMI	15–38.6 (21.39)	15–38.6 (21.63)	15.8–29.1 (21.23)

Written informed consent was obtained from all patients in accordance with the Declaration of Helsinki. This study was approved by the Institutional Review Board on Bioethics and Biosafety of BGI (BGI‐IRB, Shenzhen 518083, China) with approval number BGI‐IRB 15079.

### Comparisons of phenotypes and biochemical levels

Body mass index (BMI), age, clinical indices, and plasma levels of VB12 were compared between the *H. pylori*‐positive and *H. pylori*‐negative groups using the Wilcoxon test. The gender ratio of each group was compared using the chi‐square test.

### Metagenomic sequencing

Fecal DNA was extracted following the MetaHIT protocol 17, and then, single‐end metagenomic sequencing was performed using the BGISEQ‐500 platform. The low‐quality reads were discarded, and the host DNA was removed based on the human reference genome hg19 using SOAP2.22 18 (identity ≥ 0.9).

### Profile generation

The clean reads were mapped to the integrated gene catalog (IGC, http://meta.genomics.cn) using SOAP 2.22 (identity ≥ 0.95) 19, relative gene abundance profiles were produced; then, the species, genus, KEGG module, and pathway relative abundance profiles were generated, according to the Integrated Microbial Genome reference database (IMG, https://img.jgi.doe.gov/) 20.

### Rarefaction analysis

Rarefaction analysis was performed to assess the gene richness of *H. pylori*‐positive and *H. pylori*‐negative samples. For a given number of samples, we performed random sampling 100 times in the cohort with replacement and estimated the total number of genes that could be identified from these samples.

### Enterotype and diversity analysis

The enterotype of each stool sample was analyzed using a PAM‐based method on genus profiles 17, 21. The Shannon index was used to determine within‐individual alpha‐diversities in gene, species, genus, and phylum level. The Bray–Curtis distance, calculated in r 3.4.2 (vegan package) 22, was used to estimate the between‐individuals beta‐diversity at the gene level.

### Metagenome‐wide association study and metagenomics linkage group‐based analysis

The marker genes that were significantly different in relative abundance between the *H. pylori*‐positive and *H. pylori*‐negative groups were identified using the Wilcoxon test. The marker genes were then clustered into metagenomics linkage groups (MLGs) according to their abundance variation across all samples. The taxonomic assignment and abundance profiles of the MLGs were then obtained 17, 23. The MLGs with gene cluster number ≥ 100 were selected. To identify confounding factors, we first calculated correlations of MLG abundances with age, BMI, and gender. Wilcoxon test would be used for MLGs which are not correlated with any one of the three factors, to compare the abundance between HPI‐positive and HPI‐negative groups. Otherwise, logistic model would be used to remove confounding effects. The co‐presented relationships among the MLGs were calculated using Spearman's rank correlation and visualized in Cytoscape 3.6.0.

A classification model using MLG abundance profiles in gut microbiome was established for the prediction of HPI status. Tenfold cross‐validation was performed, based on a random forest model (r 3.4.2, randomForest 4.6‐10 package 24). The training set consisted of 250 randomly selected samples (152 negative and 98 positive) from a total of 313 samples, and the test set was comprised of the remaining 63 samples (33 negative and 30 positive). The performance of the classifier was assessed by ROC curve separately in the training set and test set. The probability of being *H. pylori*‐positive was calculated, and the ROC curve was drawn using the pROC package 24 for r 3.4.2.

### KEGG analysis

Differentially enriched KEGG modules were identified according to their reporter scores 23. A reporter score of Z ≥ 1.6 (90% confidence according to a normal distribution) was used as the detection threshold for significantly differentiating modules.

## Results

### Overview of gut microbiome

**Figure 1:**
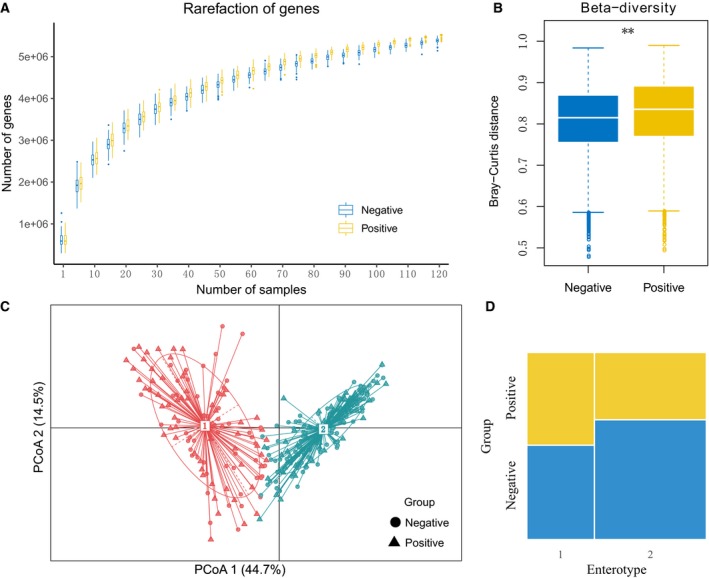
Overall observations of gut microbiome. (A) The rarefaction curves of gut microbial genes in *H. pylori*‐negative and *H. pylori*‐positive group. (B) Between‐group β‐diversity of the two groups on gene level (*P* < 0.01, Wilcoxon test). (C) The principal coordinate analysis (PCoA) plot showing the similarity of microbial composition of 313 fecal samples. (D) The mosaic plot showing the proportion of each type of sample, blue and yellow represent HPI‐negative and HPI‐positive group separately (*P* < 0.05, χ^2^ test).

To determine whether HPI accompanies with changes in the gut microbiome, metagenomic sequencing was performed on 313 stool samples from volunteers, of which 128 were *H. pylori*‐positive, and 185 were *H. pylori*‐negative (Table 1). The distribution of BMI and age in the HPI‐negative and HPI‐positive groups is shown in Fig. S1, and there was no significant difference in BMI and age between the two groups, whereas there was a significant difference (χ^2^ test, *P* < 0.05) in gender ratio between the two groups. The shotgun metagenomic sequencing was carried out using the BGISEQ‐500 platform and generated an average of 42 million single‐end reads per sample. To ensure that our sample size was sufficient for this study, rarefaction analysis was carried out for the *H. pylori*‐positive and *H. pylori*‐negative groups, and the results showed that the gene richness approached saturation in each group and showed higher richness in the *H. pylori*‐positive group (Fig. 1A). Referring to previous human gut microbiome metagenomic studies, we determined that the sample size in our study was sufficient 17, 23, 25.

The richness and diversity of the gut microbiota in the two groups were investigated. In contrast to the results of stomach microbial communities, there was no significant difference in the gene richness, species richness, and the within‐sample diversity between the *H. pylori‐*positive and *H. pylori*‐negative groups (Wilcoxon test, *P* > 0.05; Fig. S2). On the other hand, at the gene level, the intersample diversity was significantly higher in the *H. pylori*‐positive group compared with the *H. pylori*‐negative group (Wilcoxon test, *P* < 0.05; Fig. 1B). Enterotype is also a general characteristic of the human gut microbiota 21, 23, 26. We divided the samples into two enterotypes (or clusters) using a PAM‐based method; the *H. pylori‐*positive and *H. pylori*‐negative groups both contained two enterotypes (Fig. 1C). However, the *H. pylori‐*positive group had a higher proportion of enterotype 1, which contains a high level of *Prevotella,* whereas the healthy *H. pylori‐negative* group had a higher proportion of enterotype 2, which is dominated by *Bacteroides* (χ^2^ test, *P* < 0.05; Fig. 1D).

### Metagenome‐wide association study and metagenomics linkage group‐based analysis

**Figure 2:**
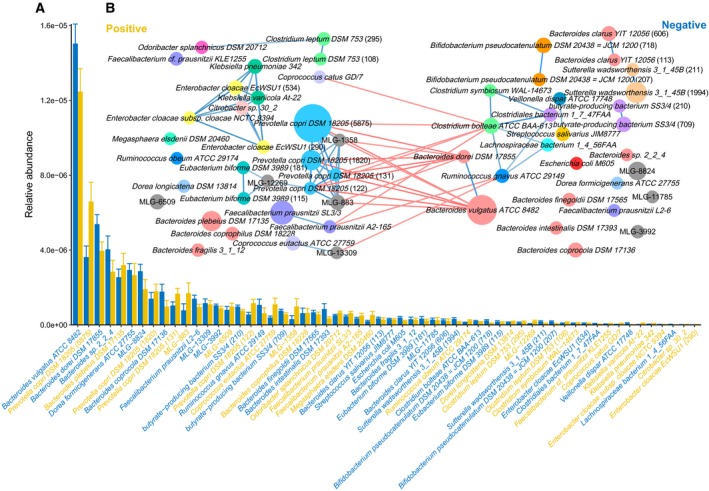
Differential MLGs with gene number ≥ 100. (A) The mean relative abundance of the MLGs in two groups (error bars represent standard error of the mean, blue represents MLG enriched in negative group, and yellow represents MLG enriched in positive group). (B) The co‐occurrence network of the MLGs, the nodes represent the MLGs with the species name or id displayed in the center, the size of the nodes indicated the gene number within the MLG, the connecting lines depict the Spearman correlation coefficient values above 0.4 (blue) or below −0.4 (red).

In order to investigate the HPI‐related signatures of the gut microbiome, we adopted the MLG method to cluster the genes with significant abundance differences into taxa. We identified 189,771 genes that were differentially enriched in *H. pylori*‐positive or *H. pylori*‐negative groups (adjusted *P* < 0.1, Wilcoxon test), approximately 1.9% of the total gene numbers in the integrated gene catalog (IGC). Approximately 56.3% and 43.7% of the gene markers were significantly enriched in the *H. pylori*‐positive group and *H. pylori*‐negative group, respectively. There were 58 MLGs that had significantly different relative abundances between the *H. pylori*‐positive and *H. pylori*‐negative groups (adjusted *P* < 0.05, Wilcoxon test; Fig. 2A,B; Tables S2 and S3). Among the differential MLGs, 31 were enriched in the *H. pylori*‐positive group and 27 were enriched in the *H. pylori*‐negative group.

We observed that the MLGs that were annotated as *Prevotella copri*, which is a proinflammatory bacterium and has been shown to correlate with the onset and development of rheumatoid arthritis (RA) 25, were enriched in *H. pylori*‐positive individuals. In addition, the relative abundances of *Enterobacter cloacae* and *Klebsiella pneumoniae,* two infectious pathogenic bacteria that can cause bacteremia and septicemia commonly associated with hospital infections 27, were clustered together and also enriched in the *H. pylori*‐positive group. The relative abundances of *Sutterella wadsworthensis*,* B. vulgatus*, and *E. coli* were significantly higher in the *H. pylori*‐negative group compared with the *H. pylori*‐positive group. In contrast to *P. copri*,* S. wadsworthensis* has been found to be enriched in people without RA 25. Additionally, the *P. copri* cluster that was enriched in the *H. pylori*‐positive group negatively correlated with *H. pylori*‐negative‐enriched MLGs (Fig. 2B), indicating that, in *H. pylori*‐positive individuals, the gut microbial network shifts via interspecies interactions.

**Figure 3:**
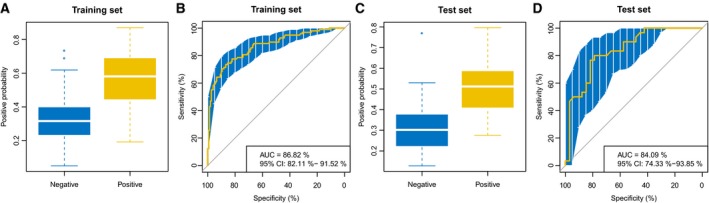
The positive probability of the samples and receiver operating curve (ROC) of the classifier in the training data and test data. (A) ROC of the classifier in the training data. (B) Positive probability of samples in the test data. (C) Positive probability of samples in the training data. (D) ROC of the classifier in the test data.

To evaluate the possibility of determining HPI using gut microbial markers, we constructed a classifier based on the random forest model. The validated optimal model selected 30 MLG markers, and the area under the ROC was 86.82% in the training set and 84.09% in the test set (Fig. 3A–D), indicating that gut microbial MLGs can be used to classify whether a subject has been infected with *H. pylori*.

### KEGG analysis

**Figure 4:**
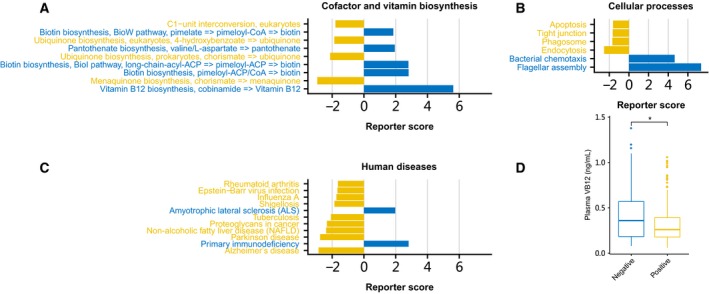
Microbial function and plasma VB12 level differences between *H. pylori*‐negative and *H. pylori*‐positive groups. (A–C) The function reporter score bar plot of cofactor and vitamin biosynthesis, cellular processes, and human diseases. (D) Differences in the level of plasma VB12 between *H. pylori*‐negative and *H. pylori*‐positive groups (*P* < 0.05, Wilcoxon test).

To explore the association between the HPI and alterations in human gut microbiome function, the microbial genes were mapped to KEGG modules and pathways; the KEGG pathways and modules with levels significantly different between the *H. pylori‐*positive and *H. pylori*‐negative groups are listed in Tables S4 and S5. The genes with functions related to cofactor and vitamin biosynthesis, cellular processes, and human diseases were enriched in different groups (Fig. 4A–C). In particular, the level of KEGG modules for VB12 biosynthesis was significantly diminished in the *H. pylori*‐positive group (Fig. 4A). After then, comparison of the plasma VB12 concentrations between the two groups revealed that the *H. pylori*‐positive group had significantly lower VB12 levels than the *H. pylori*‐negative group (Fig. 4D), indicating that infection with *H. pylori* may increase the risk of VB12 deficiency. The differential genes assigned to KEGG pathways involved in flagella assembly and bacterial chemotaxis were diminished in the HPI‐associated microbiome (Fig. 4B), indicating the weaker mobility and chemotaxis of intestinal microbes in *H. pylori*‐positive individuals. Additionally, among the 11 enriched disease‐related KEGG pathways, nine were enriched in the *H. pylori*‐positive group, of which four were related to infectious diseases (Fig. 4C).

## Discussion

The current study identified HPI‐related gut microbiome alterations and provided a catalog of changes for further research. In addition, a classifier to discriminate HPI status using microbial markers was constructed and evaluated. Further analyses on microbial functional profiles and metabolite level comparisons, revealed the diminished level of blood VB12 and decreased microbial VB12 biosynthesis capacity in HPI‐positive individuals, indicating that HPI‐related VB12 deficiency could be caused by imbalance of the gut microbiome.

The relative abundance of MLGs, annotated as *P. copri,* was significantly enriched in *H. pylori*‐positive individuals. *P. copri* is an immune‐relevant gut microbe. It has been reported that the continuous colonization of *H. pylori* in the stomach induces the host immune response 4, 10. There is a complex interaction between the intestinal microbes and the host; gut microbial communities are crucial for host immune system function, and in turn, the immune environment affects the gut microbial structure 28. *P. copri* is very prosperous in a proinflammatory gastrointestinal environment and further increases the level of inflammation 29. RA, which is a prevalent systemic autoimmune disease, has been shown to associated with gut microbiome dysbiosis, and *P. copri* is associated with the onset and severity of rheumatoid arthritis 25, 30. The enrichment of *P. copri* in *H. pylori*‐positive individuals may correlate with the change of intestinal immune environment.

We observed that the VB12 biosynthesis module was depleted in the *H. pylori*‐positive group, and plasma VB12 concentration was also significantly lower in the *H. pylori*‐positive group compared with the *H. pylori‐*negative group. Biologically, VB12 is a type of cobalt corrinoid, and as humans and most other animals are incapable of VB12 production, it is exclusively produced by the microorganisms, particularly anaerobes 31. Substantial previous epidemiological studies have revealed that HPI correlates with lower VB12 levels and even VB12 deficiency 6, 7. Our current results suggest that the HPI‐related dysbiosis of intestinal microbiota can affect the production of VB12 in the human intestine. Previous research infers that gastric sinusitis, caused by HPI, may progress to type B chronic gastritis, accompanied by decreased gastric acid secretion, which leads to malabsorption of VB12 7, 32. Therefore, both the production and absorption capacity of VB12 can be weakened by HPI, increasing the risk of VB12 deficiency.

There are some limitations to this study that need to be addressed. The effects of HPI on the host have individual and population variations, and strain specificity. These differences need to be addressed in subsequent studies with a larger sample size. Further studies should also include host proteomics, metabolomics, and other data for multiomics integrated analysis, which could then furtherly reveal the mechanisms by which *H. pylori* impacts the host.

In conclusion, our study sequenced 313 fecal samples using the metagenomic shotgun method and analyzed the differences between *H. pylori*‐positive and *H. pylori*‐negative groups. It shows that HPI is associated with changes in human intestinal microbial composition and function in the Chinese population. Also, the abundance of the immunologically related bacteria *P. copri* was significantly different between the two groups. In the *H. pylori*‐positive group, the levels of flagella assembly and bacterial chemotaxis‐related pathways were significantly depleted, and these intestinal changes in the *H. pylori*‐positive group may have further effects on the host. The lower level of VB12 biosynthesis module was associated with the lower VB12 concentrations in the blood of *H. pylori*‐positive individuals, indicating that HPI‐related gut microbiota dysbiosis can increase the risk of VB12 deficiency. Our study provides new clues into the interactions between *H. pylori* and host gastrointestinal microecology.

## Conflict of interest

The authors declare no conflict of interest.

## Author contributions

DW designed the study. XZ, HY, XL, and RW managed the project. YZ, YL, and DW contributed to the acquisition of sample and clinical data. DW, QD, YL, ST, and QW performed data analyses. DW wrote the paper. DW, YL, and ZH revised the paper. All authors approved the final paper.

## Supporting information


**Fig. S1.** Distribution of BMI and age. (a) Distribution of age. (b) Distribution of BMI.

**Fig. S2.** Alpha diversity in gene, species, genus, and phylum levels. (a) Alpha diversity in gene level (b) Alpha diversity in species level. (c) Alpha diversity in genus level. (d) Alpha diversity in phylum level.

**Table S1.** Phenotype of all volunteers.
**Table S2.** The information for all MLGs containing more than 100 genes.
**Table S3.** The profile of MLGs contains more than 100 genes.
**Table S4.** Differentially enriched KO pathways between *H. pylori* positive and negative group (reporter score of ≥1.6).
**Table S5.** Differentially enriched KO modules between *H. pylori* positive and negative group (reporter score of ≥1.6).
