# Supplementary figures and images for: Alterations in the human gut microbiome associated with Helicobacter pylori infection
Source: FEBS Open Bio. 2019 Aug 10;9(9):1552–60. doi: 10.1002/2211-5463.12694 (PMC6724102; doi:10.1002/2211-5463.12694)

**a**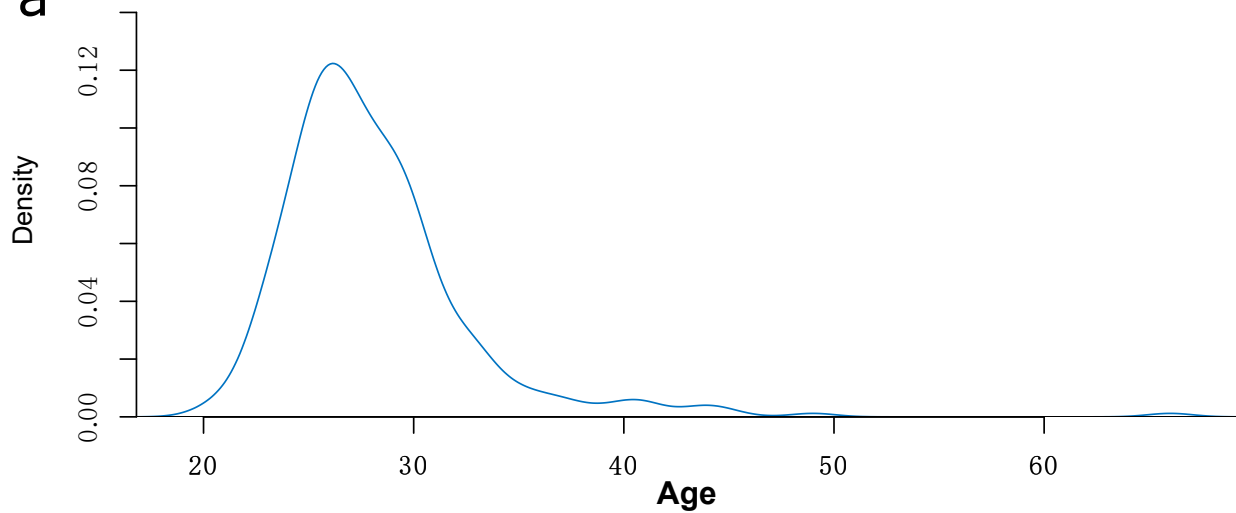**b**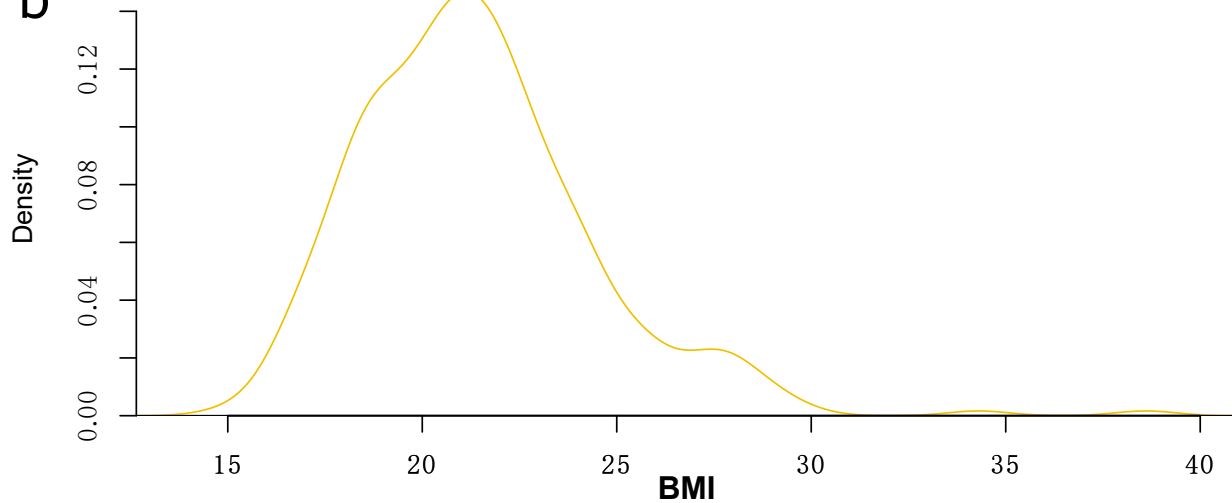

Supplement: Supplementary file 1 — Fig. S1. Distribution of BMI and age. (a) Distribution of age. (b) Distribution of BMI. [file FEB4-9-1552-s001.pdf]

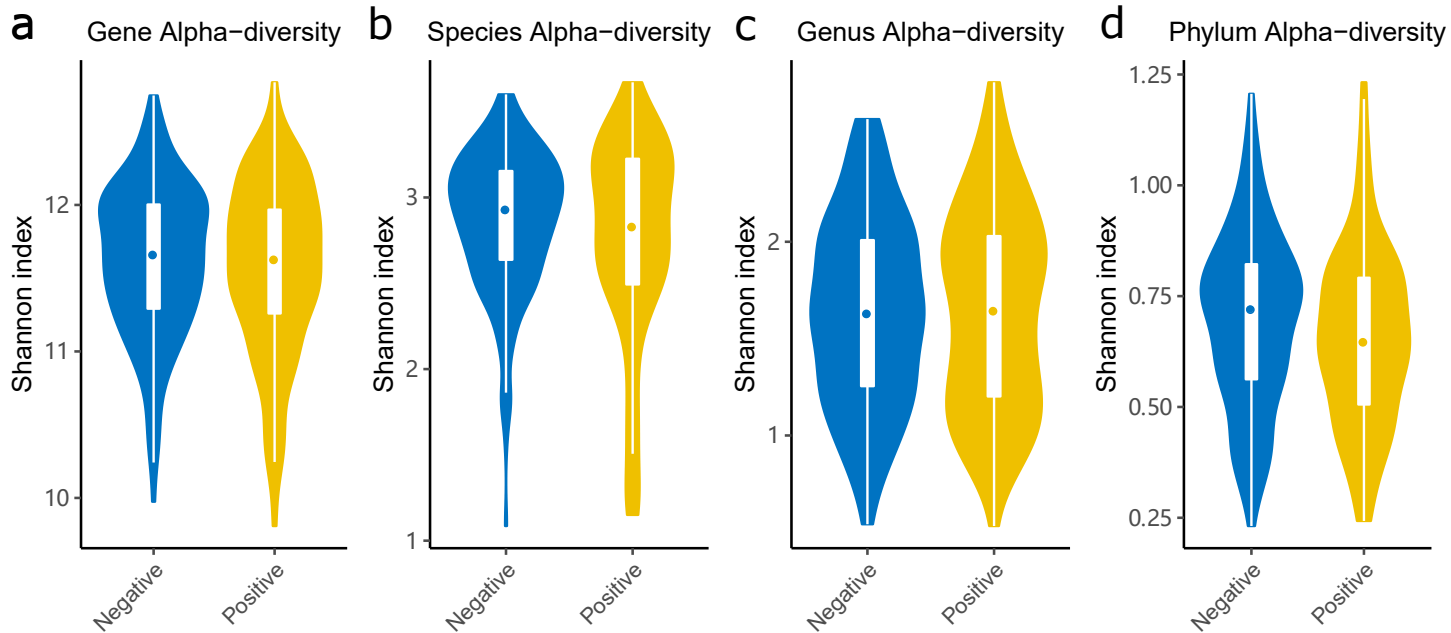

Supplement: Supplementary file 2 — Fig. S2. Alpha diversity in gene, species, genus, and phylum levels. (a) Alpha diversity in gene level (b) Alpha diversity in species level. (c) Alpha diversity in genus level. (d) Alpha diversity in phylum level. [file FEB4-9-1552-s002.pdf]
